# Use of a Specific Phage Cocktail for Soft Rot Control on Ware Potatoes: A Case Study

**DOI:** 10.3390/v13061095

**Published:** 2021-06-08

**Authors:** Eugenia N. Bugaeva, Maya V. Voronina, Dmitry M. Vasiliev, Anna A. Lukianova, Nikolay N. Landyshev, Alexander N. Ignatov, Konstantin A. Miroshnikov

**Affiliations:** 1 Research Center “PhytoEngineering” Ltd., 141880 Rogachevo, Moscow Region, Russia; bygaeva.genia@gmail.com (E.N.B.); maya.khodykina@gmail.com (M.V.V.); vasilyevdim@mail.ru (D.M.V.); an.ignatov@gmail.com (A.N.I.); 2 Shemyakin-Ovchinnikov Institute of Bioorganic Chemistry, Russian Academy of Sciences, Miklukho-Maklaya Str., 16/10, 117997 Moscow, Russia; a.al.lukianova@gmail.com (A.A.L.); swarekwood@gmail.com (N.N.L.); 3 Department of Biology, Lomonosov Moscow State University, Leninskie gory, 1, bldg. 12, 119234 Moscow, Russia; 4 Institute of Medicine, RUDN University, Miklukho-Maklaya Str., 8, 117198 Moscow, Russia; 5 Agrobiotechnology Department, Agrarian and Technological Institute, RUDN University, Miklukho-Maklaya Str., 6, 117198 Moscow, Russia

**Keywords:** potato (*Solanum tuberosum*), soft rot, *Pectobacterium*, bacteriophage, phage control

## Abstract

Using bacteriophages (bacterial viruses) to control pathogenic bacteria is a promising approach in horticulture. However, the application of this strategy in real conditions requires compliance with particular technological and environmental restraints. The presented paper concerns the process of phage selection to create a cocktail that is efficient against the circulating causal agents of potato soft rot. The resulting phage cocktail causes a complete lysis of a mixture of circulating pectobacterial strains in vitro. In the context of being used to treat ware potatoes during off-season storage, the protocol of phage application via the humidity maintenance system was designed. The phage cocktail was shown to reduce the population of *Pectobacterium* spp. 10–12-fold, achieving a population that was below a symptomatic threshold.

## 1. Introduction

The potato (*Solanum tuberosum*) is one of the most important staple food crops across the world. Because of specific biologic properties of the crop and intensive agriculture, many fungal, viral, and bacterial diseases inflict significant losses to vegetating and ware potatoes. Soft rot caused by bacteria belonging to *Pectobacterium* and *Dickeya* genera (soft rot Pectobacteriaceae (SRP)) may result in losses of up to 30% of the annual potato yield [1]. The application of pesticides and chlorine compounds is strictly limited for food tubers; therefore, there is a lack of efficient methods for soft rot control for stored potatoes. The use of bacteriophages (phages) to combat pathogenic bacteria is a promising strategy. Mixtures of phages specific to the target bacteria (phage cocktails) have been successfully used for the control of various bacterial phytopathogens (reviewed in [2,3,4]). In such cases, the use of phage cocktails can significantly reduce crop spoilage and corresponding economic losses. It is important to note that, currently, bacteriophages are generally recognized as being safe by the US Food and Drug Administration regarding the food sector. Moreover, post-harvest application of *Pectobacterium* phages is allowed in Europe [4]. Numerous attempts have been made to systematize the application of individual phages and phage cocktails to protect potatoes from soft rot [4,5,6,7,8,9,10,11]. However, most studies were limited to in vitro conditions and model strains of the pathogens. Successful realization of a phage-based control strategy requires a comprehensive understanding of the range of target pathogens, phage selection, purification and storage, application of resulting phage cocktails, and monitoring of the results. The study described here addresses some of these aspects, summarizing the prophylactic treatment of soft rot in a commercial potato warehouse during the 2019/2020 storage season.

## 2. Materials and Methods

### 2.1. Sampling the Phytopathogens

Pectolytic bacterial strains were isolated from rotting potato tubers and stems collected from farms in the northern part of the Moscow region, Russia, from May to September 2019. Initial isolation was carried out using crystal violet pectate (CVP) agar medium [12]. Cavity-forming colonies were picked up and replated on CVP agar several times, to reach a homogeneous morphologic appearance of the colonies. Further cultivation of the isolates was performed routinely at 28 °C in lysogeny broth (LB) liquid medium and on LB agar plates. For long-term storage, the liquid cultures were supplied with 10% glycerol and frozen at −80 °C. Bacterial genomic DNA was isolated using a DNA Easy Blood and Tissue kit (Qiagen, Hilden, Germany) according to the manufacturer’s instructions.

### 2.2. Differentiation of Pectobacterium spp.

The taxonomic distribution of the soft rot causative agents was assessed using diagnostic PCR tests for *Pectobacterium* spp., *Dickeya* sp., *P. atrosepticum*, *P. wasabie* (syn. *P. parmentieri*), *P. brasilense*, and *P. carotovorum* [13]. Additionally, the strains, especially those with no specific PCR response, were verified using 16S rDNA gene sequencing and the BIOLOG microbial ID system (Biolog, Hayward, CA, USA). The properties of the isolates are presented in Table 1.

### 2.3. Testing the Strains for Phage Susceptibility

The ability of phages to infect bacterial cells was tested using spot-on-lawn assays on LB agar plates [14]. In total, 50 bacteriophages previously isolated from environmental sources were used to test the specificity of the antibacterial effect. Among phages causing bacterial lysis, those revealing a broader host range were selected as ingredients of the phage cocktail. All selected phages had their genomes sequenced and presented a strictly lytic behavior with respect to their isolation strains. The detailed properties of some of these were previously reported [8,15,16]. General features of the phages comprising the experimental phage cocktail are presented in Table 2.

The phage ability to lyse host bacteria was assessed as previously described [17], using individual phage strains or a phage cocktail. Briefly, 250 mL of the broth culture of the target strain in the mid-exponential growth phase was mixed with 250 mL of the phage suspension at multiplicities of infection (MOI) of 0.01 and 500 mL of LB broth. The mixture was then incubated, with shaking, at 28 °C. Every 10 min, aliquots were taken, and the appropriate dilutions were spread on LB agar plates and incubated overnight at 28 °C. The next day, colonies were counted. Then, 250 mL of liquid culture of the same host strain was mixed with 250 mL of phage buffer (50 mM Tris-HCl (pH 7.5), 100 mM NaCl, 8 mM MgSO4), and 500 mL of LB, to observe the growth pattern of uninfected bacteria. All procedures were repeated in triplicate, and the results were averaged. 

### 2.4. Phage Propagation and Purification

The bacterial strains used for propagation of the corresponding phages are listed in Table 2. Bacterial cultures were grown to OD_600_ = 0.4 at 25 °C; then, phage suspensions were added at a multiplicity of infection (MOI) of 0.01. Further incubation was performed at 28 °C until completion of the lysis, and then chloroform was added. Bacterial debris was pelleted by centrifugation at 7000× *g* for 20 min. The phage lysate was precipitated with polyethylene glycol (PEG) 8000 (10%)–NaCl (0.6%) at 4 °C overnight, centrifuged at 8000× *g* for 20 min, and resuspended in phage buffer; then, 1 M KCl was added. The mixture was incubated on ice for 20 min and centrifuged (12,000× *g* for 20 min at 4 °C) to precipitate the PEG solution [18]. The phage titer of the resulting preparation was determined using the double-agar layer technique [17] with minor modifications. Usually, the titers of the resulting phage suspensions consisted of about 10^11^ plaque-forming units (PFU)/mL. Phage stocks were mixed and diluted with water to result in ~10^8^ PFU/mL for each phage. Alternatively, phage lysates were subjected to sterile filtration through a 0.45 μm membrane (Millipore, Burlington, MA, USA) and mixed to produce a combined phage preparation with the same activity.

### 2.5. Application of Phage Cocktails in the Warehouse

The experiment location was warehouse #41, chambers 41.1 and 41.2, of Agropark Rogachevo (geographical coordinates 56°25′28” N, 37°9′15” E). Ten liters of phage cocktail with a titer of ~10^8^ PFU/mL for each phage was applied to the moisture maintenance system and sprinkled through the chambers automatically within ~1 h. The storage conditions were 4–7 °C and 95% humidity. Treatments were repeated weekly through the storage season (a total of 24 treatments). Chambers 1.1 and 1.2 of the neighboring warehouse #1 were used as a control, without phage application (filtered tap water was used as the control).

### 2.6. Control of Phage and Bacterial Concentration in the Warehouse Environment

Samples from 30 tubers cv. La Strada were taken daily from the center and each corner of the chamber for 7 days after phage application. Tubers were peeled and potato skins were homogenized in phage buffer 1:1 (*w*/*v*). After centrifugation of the suspension and plating of the supernatant onto CVP agar, the plates were incubated for 24 h at 28 °C, and the resulting pit-forming colonies were counted. To identify phage-resistant isolates able to lyse pectate after phage treatment, the genomic DNA of such strains was isolated, and 16S rDNA was PCR-amplified and sequenced. Isolated phage-resistant strains were tested for maceration of potato tissue, using a standard potato slice method [11].

Phage PP16 was chosen for monitoring the viral population. The supernatant, after centrifugation of resuspended potato skins, was filtered through a 0.22 µm membrane (Millipore) and plated onto LB double-agar plates supplemented with indicator strain F002 (*P. versatile*). The plates were incubated for 24 h at 28 °C and phage plaques were counted. The identity of PP16 was verified by PCR, using primers 5′–CTGTCCGCAGGTAAGA and 5′–TCACTTGCCACCTAGTA, amplifying the unique sequence of gene 57 encoding PP16 tail spike protein (PCR conditions 20 cycles: 94 °C × 40 s + 57 °C × 40 s + 72 °C × 40 s).

Experiments were repeated five times through the storage season, and the results were averaged.

### 2.7. Statistical Analysis

Statistical analysis of the data was performed in RStudio 4.0.3 (Rstudio, Boston, MA, USA). The statistical difference between treatment and control was estimated using a Mann–Whitney U-test on average values across the whole length of the experiment. We compared the difference between values in triple probes with repeatability limit calculated using Pearson’s coefficient (*p* = 0.95) to assess the data repeatability.

## 3. Results

### 3.1. Phytopathogens Causing Soft Rot of Potato in Northern Moscow Region in 2019

Potato tubers affected by soft rot were collected in the area surrounding the future point of potato storage. Taking into account the limited ability of SRP for independent long-range spread, it is presumed that these pathogens present an immediate danger to stored potatoes. Extensive testing revealed strains of *P. brasilense* (Pbr), *P. polaris* (Ppo), and *P. versatile* (Pve) to be the most common bacterial pathogens causing soft rot (Table 1). All strains initially defined as *P. carotovorum* were specified as Pve by 16S rDNA sequencing. *P. versatile* (syn: *P. maceratum*) is a species that was recently separated from *P. carotovorum* due to distinct genomic features [19,20]. Generally, this set of species is typical for SRP populations in Central and Eastern Europe [21,22,23]. No representatives of *Dickeya* spp. and *P. atrosepticum* (Pat), which resulted in severe outbreaks of soft rot in the 2000s, and *P. parmentieri* (Ppa), which recently emerged as an abundant potato pathogen [24,25], were detected. The diversity of the isolated species was more correlated to the cultivar and seed lot of potato, rather than to geographic location or the particular weather conditions of the growing season. This observation supports the common opinion that contaminated seed potato is a major source for soft rot spreading [1]. It is worth noting that diseases caused by oomycetes and fungi, particularly *Phytophtora infestans* and *Fusarium* spp., were identified as being typical during the 2019 agricultural season in Central Russia. Bacterial diseases were rare, and it was hard to distinguish the pectolytic isolate as a secondary cause of disease after fungal or oomycete infection.

### 3.2. Construction of the Pathogen-Adapted Phage Cocktail

**Figure 1:**
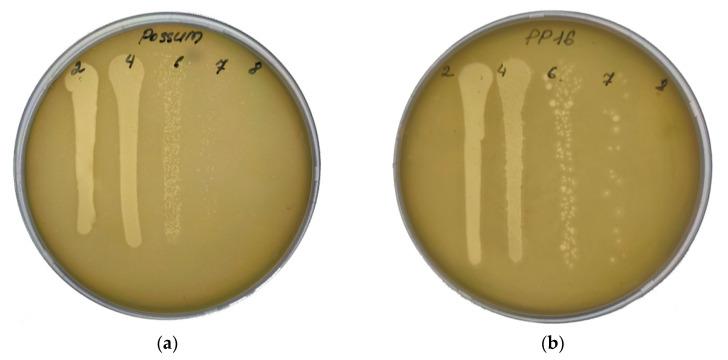

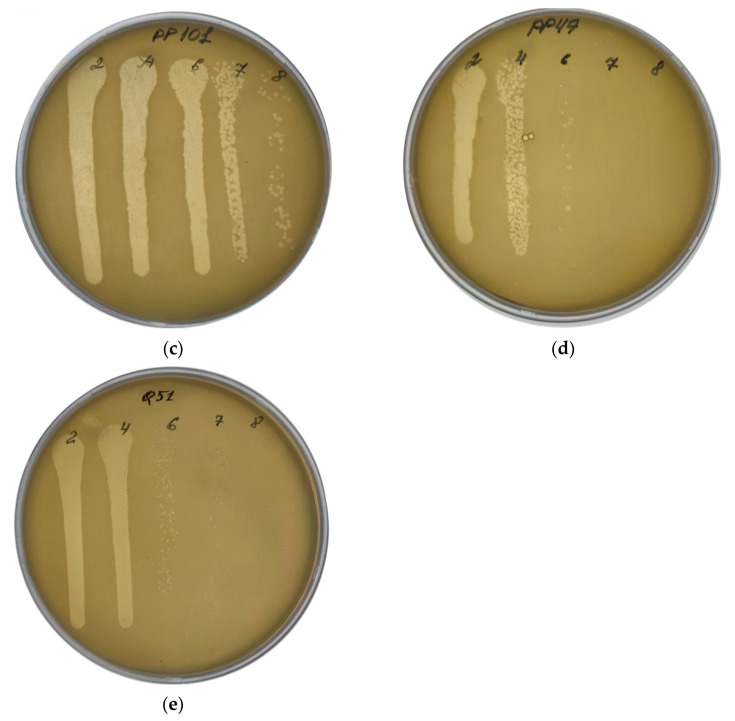
Plaques formed by bacteriophages comprising the phage cocktail. Each phage was applied in different concentrations to the corresponding propagation host. The numbers indicate the dilution of the phage lysate (10-fold dilutions): (**a**) Possum/F041; (**b**) PP16/F002; (**c**) PP101/F152; (**d**) PP47/F157; (**e**) Q51/F018.

**Figure 2:**
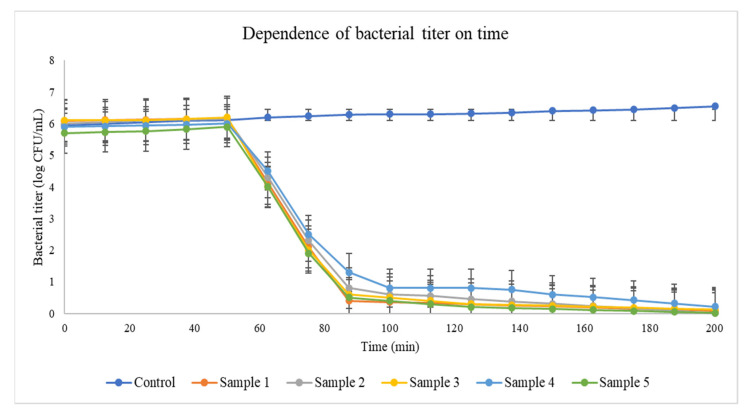
Bacteriolytic effect of phage cocktail on pectobacterial strains *in vitro*. The mixture of phages shown in Table 2 (MOI = 0.01 for each phage in all experiments) was applied to Pod 2_2 (Control), Pve 2_1 (red), Pbr 4 (gray), Pve 6_2 (yellow), Ppo10 (blue), and Pbr 9_1 (green).

Except for a single strain of *Pectobacterium odoriferum* (Pod), all isolated pathogens causing soft rot were susceptible to one or several bacteriophages from the panel of tested phages. Considering general requirements for therapeutic phages such as lytic infection cycle, broad infection range, comprehensive genomic and biological characterization, and high yield in cultivation [2,3,26,27], five phages were selected for the phage cocktail for further application (Figure 1, Table 2). The set of five bacteriophages effectively reduced *in vitro* 18 of the 19 bacterial strains from the population isolated in 2019 (Figure 2). The bacteriophages belonging to different morphologic and taxonomic groups reduced the probability of the appearance of phage-resistant mutants in the population of pathogenic bacteria. Despite the fact that the total elimination of target bacteria was never achieved *in vitro*, no substantial growth of phage-resistant bacteria or their enhanced pectolytic activity was observed, supporting previous experimental evidence [27,28].

Selected phages were cultivated at a preparative scale using the corresponding characterized and sequenced host strains (Table 2). Both types of preparation (immediately mixed sterile filtered lysates and stored PEG-purified phage stocks with adjusted concentration) were stable at 4 °C for 1 month, losing less than one order of titer activity and exhibiting no bacterial contamination. For long-term storage and for commercial applications, the phage cocktails should be stabilized and adapted for ambient temperature (reviewed in [29]). However, in the presented case, phage cocktails were applied within 1 week of production, and this critical point was beyond the scope of the study. 

### 3.3. Construction of the Pathogen-Adapted Phage Cocktail

The facilities of Agropark Rogachevo use conventional conditions for potato storage. Ware potatoes are stored at 4–7 °C, with a constant relative humidity of about 95% provided. These conditions retard the growth of mesophilic bacteria. Periodically, potatoes are shuffled to prevent the formation of anaerobic cavities accelerating soft rot within the chambers. Therefore, the phage treatment mostly has a preventive purpose. The lifetime of phages on the surface of potato tubers was probed, and changes in phage concentration reflecting the existence of host bacteria were monitored.

It was revealed that the best option for phage application is the use of humidity maintenance system sprinklers. In this case, the finely dispersed droplets of water containing phages did not condense on the surface of the bulk-stored potatoes but penetrated deep into the bulk. After each application, we observed the concentration of phage PP16 to be maintained stable at 10^4^–10^5^ PFU/tuber for 1 week, before gradually lowering later. The stability of phages in favorable conditions (low temperature, normal humidity, the absence of UV radiation) corresponds to the results of previous studies [11]. No local bursts of phage concentration following phage infection were detected.

**Figure 3:**
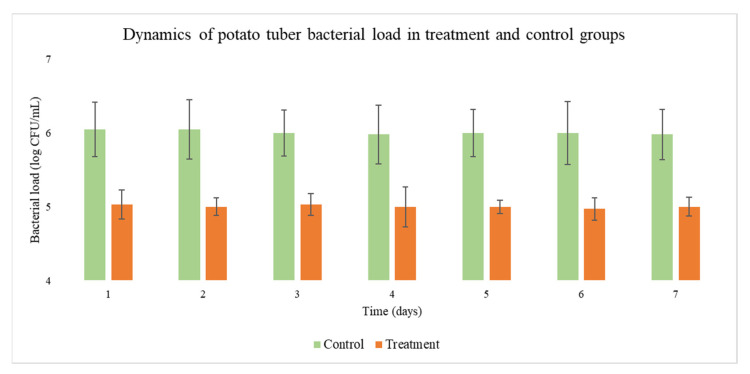
Reduction in the population of pectolytic bacteria on the surface of potato tubers after phage application. Tubers treated with phage cocktail compared to treatment with water. Error bars indicate standard deviation (control); *p* < 0.05 for averaged values comparison across the whole experiment.

The treatment of ware potatoes with the phage cocktail resulted in a 10- to 12-fold reduction in the population of pectolytic bacteria on the tubers (Figure 3). According to the statistical analysis, the difference between treatment and control groups reached *p* < 0.05. Furthermore, the differences between the median values of triplicate probes were lower than the corresponding repeatability limit (*p =* 0.95), which supports the choice of method. However, the averaged concentration of such bacteria on the surface of control tubers was also low. Therefore, no outbreaks of soft rot were detected in a control warehouse where the potatoes were stored in conventional conditions without phage treatment. This observation raises an important question about the concentration of bacteria causing symptomatic soft rot and the corresponding concentration of phages sufficient to quench the infection. No definite answer is available. Most in vitro studies used a 10^6^–10^7^ CFU load of bacteria to cause reproducible and fast infection of injured potato tuber tissues (e.g., tuber slices) under conditions favoring bacterial growth. It is possible that potato tubers with intact skin become diseased under a higher bacterial load, and conventional storage conditions prevent bacterial growth above this threshold, even if the latent infection of ware tubers is permanently present. In this case, phage treatment would be a complementary measure without an immediate or noticeable effect. However, if the bacterial load on ware potatoes is above the infection threshold, the prophylactic effect of phage control may be significant.

### 3.4. Probing the Phage-Resistant Bacteria Causing Soft Rot

Several cases of minor soft rot of the tubers treated with the phage cocktail were registered after the ware potatoes were packaged and sent to retail stores, where they were kept on shelves at ambient temperatures. Moreover, a residual population of pectolytic bacteria was detected on the surface of the tubers, even after repeated phage treatment. The study of phage-resistant strains and causal agents of soft rot symptoms demonstrated that these bacteria exhibit pectolytic activity on CVP media and cause tissue maceration of potato slices at 28 °C. The sequencing of 16S rDNA genes revealed that the isolates belonged mostly to SRP resistant to the applied phages (*P. polaris* and *P. carotovorum*) and to bacteria of different taxa possessing pectolytic activity (Table 3). 

After the recognition of several clonal groups of *P. carotovorum* as a new species and the elevation of its subspecies to the species level, the strains attributed as *P. carotovorum* have rarely been reported as primary causative agents of potato soft rot disease. Therefore, a weak pathogenic strain could be overlooked in sampling, and no specific phages were available in the applied cocktail. *P. polaris* is a recently established species [30]; thus, the diversity of its strains is understudied. Other identified bacteria are not normally linked to soft rot in potatoes. *Pseudomonas moraviensis* (isolate 7523) is a common soil and rhizosphere bacterium known for its role in xenobiotic pollutant destruction [31]. Although *Cryseobacterium* spp. (isolate 7413) are also known to be part of the rhizospheric and plant endophytic bacterial community [32], its phytopathogenic potential has not been studied. *Pantoea agglomerans* (isolate 7231) is a well-known phytopathogen for onions and mushrooms, but not for potatoes. However, a number of reports on soft rot in potatoes caused by bacteria outside the SRP group have been published [1]. Therefore, the importance of such pathogens should be carefully evaluated, especially when the population of SRP strains is inhibited. 

## 4. Conclusions

The use of bacteriophages, i.e., specific viruses infecting bacteria, is a promising strategy for the control of bacterial pathogens in medicine, the food industry, and agriculture. The successful implementation of phage control requires solutions to many questions dealing with the genomics of pathogenic bacteria and their corresponding phages, the molecular biology of their interaction, and issues to be resolved in the construction, production, application, and regulatory registration of phage preparations or cocktails. The work presented addresses some of these problems. The preliminary study of the pathogens addressed made it possible to construct a cocktail comprising a limited number of diverse phages that effectively reduced the population of bacteria without substantial evolution of phage-resistant mutants. The application of the phage cocktail using a humidity maintenance system provided an even distribution and stability of phages inside the treated warehouse and prevented the development of soft rot caused by target pathogens. The results of the study were not completely conclusive because of the substantial efficacy of conventional precautionary measures for potato storage. This study suggests the construction of a convenient and fast method for monitoring the phage population during application and highlights the problem of bacteria not belonging to SRP causing soft rot in potatoes.

## Figures and Tables

**Table 1:** Characteristics of bacteria causing soft rot of potato in agricultural season 2019 in Moscow region.

Isolate	Potato cv	CVP	Maceration	Species	PP16	PP47	PP101	Q50	Possum
2_1	Bellarosa	+ ^1^	+	Pve	+	+	−	−	−
2_2	Bellarosa	+	+	Pod	−	−	−	−	−
2_3	Bellarosa	+	+	Pve	+	+	−	−	−
2_4	Bellarosa	+	+	Pve	+	+	−	−	−
2_5	Bellarosa	+	+	Pve	−	+	+	+	−
4	Lady Claire	+	+	Pbr	+	−	+	+	−
4_82	Gala	+	+	Pbr	−	+	+	+	−
5_1	Gala	+	+	Pbr	−	+	+	+	−
5_2	Gala	+	+	Pbr	−	+	+	+	−
6_1	Arsenal	+	+	Pve	+	+	+	−	−
6_2	Arsenal	+	+	Pve	+	+	+	−	−
8_1	Isle of Jura	+	+	Pve	+	+	−	+	+
8_2	Isle of Jura	+	+	Pve	+	+	−	+	+
9_1	Molly	+	+	Pbr	+	−	+	+	−
9_2	Molly	+	+	Pbr	+	−	+	+	−
11_1	Isle of Jura	+	+	Ppo	−	−	−	+	−
11_2	Isle of Jura	+	+	Pve	−	−	−	−	+
19	Gala	+	+	Ppo	−	−	−	+	−
20	Gala	+	+	Ppo	−	−	−	+	−

^1^ + positive result in CVP pit formation, potato tissue maceration, and plaque formation on the bacterial lawn. Pbr—*Pectobacterium brasiliense*, Pod—*P. odoriferum*, Ppo—*P. polaris*, Pve—*P. versatile*.

**Table 2:** Properties of bacteriophages comprising the experimental phage cocktail.

Phage	Morphology ^1^	Genus	Propagation Strain ^2^	Infection Range	Genome NCBI Accession Number	Genome, bp	Reference
PP16	P	Kotilavirus	Pve F002	Pve + Pbr	NC_031068	44,268	[8]
PP47	P	Pektosvirus	Pbr F157	Pbr + Pve	NC_047801	40,844	[15]
PP101	M	Suwonvirus	Pbr F152	Pbr + Pve	NC_047791	53,333	[16]
Q51	M	Kolesnikvirus	Pve F018	Ppo + Pve + Pbr	MK_053931	84,303	This work
Possum	P	Enquatrovirus	Pve F131	Pat + Pve	MN_812691	73,090	This work

^1^ P—*Podoviridae* (short, non-contractile tail); M—*Myoviridae* (long, contractile tail). ^2^ Pat—*Pectobacterium atrosepticum*, Pbr—*P. brasiliense*, Pod—*P. odoriferum*, Ppo—*P. polaris*, Pve—*P. versatile*.

**Table 3:** Phage-resistant strains of pectolytic bacteria.

Isolate	CVP Pit Formation	Maceration of Potato Slices	Species
7211	+	+	*P. carotovorum*
7231	+	+	*Pantoea agglomerans*
7232	+	+	*P. polaris*
7233	+	+	*P. polaris*
7241	+	+	*P. polaris*
7242	+	+	*P. polaris*
7243	+	+	*P. carotovorum*
7413	+	+	*Chryseobacterium* sp.
7522	+	+	*P. carotovorum*
7523	+	+	*Pseudomonas moraviensis*

## Data Availability

Genome sequences of the strains used for propagation of phages were deposited to NCBI GenBank (accession numbers F002-PDVY00000000, F018-PDVV00000000, F131–CP065030, F152-PJDM00000000, and F157-PJDL00000000). Some strains were deposited to the Russian Collection of Microorganisms (VKM) and are available as V-3419 (F018), V-3418 (F131), and V-3424 (F152).

## References

[1] Charkowski A.O. (2018). The changing face of bacterial soft-rot diseases. Annu. Rev. Phytopathol..

[2] Svircev A., Roach D., Castle A. (2018). Framing the future with bacteriophages in agriculture. Viruses.

[3] Buttimer C., McAuliffe O., Ross R.P., Hill C., O’Mahony J., Coffey A. (2017). Bacteriophages and bacterial plant diseases. Front. Microbiol..

[4] Holtappels D., Fortuna K., Lavigne R., Wagemans J. (2021). The future of phage biocontrol in integrated plant protection for sustainable crop production. Curr. Opin. Biotechnol..

[5] Carstens A., Djurhuus A., Kot W., Jacobs-Sera D., Hatfull G., Hansen L. (2018). Unlocking the potential of 46 new bacteriophages for biocontrol of *Dickeya solani*. Viruses.

[6] Lim J.A., Jee S., Lee D.H., Roh E., Jung K., Oh C., Heu S. (2013). Biocontrol of *Pectobacterium carotovorum* subsp. *carotovorum* using bacteriophage PP1. J. Microbiol. Biotechnol..

[7] Carstens A.B., Djurhuus A.M., Kot W., Hansen L.H. (2019). A novel six-phage cocktail reduces *Pectobacterium atrosepticum* soft rot infection in potato tubers under simulated storage conditions. FEMS Microbiol. Lett..

[8] Voronina M.V., Bugaeva E.N., Vasiliev D.N., Kabanova A.P., Barannik A.P., Shneider M.M., Kulikov E.E., Korzhenkov A.A., Toschakov S.V., Ignatov A.N. (2019). Characterization of *Pectobacterium carotovorum* subsp. *carotovorum* bacteriophage PP16 prospective for biocontrol of potato soft rot. Microbiology.

[9] Zaczek-Moczydłowska M.A., Young G.K., Trudgett J., Plahe C., Fleming C.C., Campbell K., O’Hanlon R. (2020). Phage cocktail containing Podoviridae and Myoviridae bacteriophages inhibits the growth of *Pectobacterium* spp. under in vitro and in vivo conditions. PLoS ONE.

[10] Adriaenssens E.M., van Vaerenbergh J., Vandenheuvel D., Dunon V., Ceyssens P.J., de Proft M., Kropinski A.M., Noben J.P., Maes M., Lavigne R. (2012). T4-related bacteriophage LIMEstone isolates for the control of soft rot on potato caused by “*Dickeya solani*”. PLoS ONE.

[11] Czajkowski R., Smolarska A., Ozymko Z. (2017). The viability of lytic bacteriophage ΦD5 in potato-associated environments and its effect on *Dickeya solani* in potato (*Solanum tuberosum* L.) plants. PLoS ONE.

[12] Hélias V., Hamon P., Huchet E., Wolf J.V.D.D., Andrivon D. (2012). Two new effective semiselective crystal violet pectate media for isolation of *Pectobacterium* and *Dickeya*. Plant Pathol..

[13] Humphris S.N., Cahill G., Elphinstone J.G., Kelly R., Parkinson N.M., Pritchard L., Toth I.K., Saddler G.S. (2015). Detection of the bacterial potato pathogens *Pectobacterium* and *Dickeya* spp. Using Conventional and Real-Time PCR. Methods Mol. Biol..

[14] Czajkowski R., Ozymko Z., Lojkowska E. (2014). Isolation and characterization of novel soilborne lytic bacteriophages infecting *Dickeya* spp. biovar 3 (‘D. solani’). Plant Pathol..

[15] Evseev P.V., Lukianova A.A., Shneider M.M., Korzhenkov A.A., Bugaeva E.N., Kabanova A.P., Miroshnikov K.K., Kulikov E.E., Toshchakov S.V., Ignatov A.N. (2020). Origin and evolution of studiervirinae bacteriophages infecting *Pectobacterium*: Horizontal transfer assists adaptation to new niches. Microorganisms.

[16] Lukianova A.A., Shneider M.M., Evseev P.V., Shpirt A.M., Bugaeva E.N., Kabanova A.P., Obraztsova E.A., Miroshnikov K.K., Senchenkova S.N., Shashkov A.S. (2020). Morphologically different *Pectobacterium brasiliense* bacteriophages PP99 and PP101: Deacetylation of O-polysaccharide by the tail spike protein of phage PP99 accompanies the infection. Front. Microbiol..

[17] Clokie M.R.J., Kropinski A.M. (2009). Bacteriophages Methods and Protocols, Volume 1: Isolation, Characterization, and Interactions.

[18] Colombet J., Robin A., Lavie L., Bettarel Y., Cauchie H.M., Sime-Ngando T. (2007). Virioplankton “pegylation”: Use of PEG (polyethylene glycol) to concentrate and purify viruses in pelagic ecosystems. J. Microbiol. Methods.

[19] Shirshikov F.V., Korzhenkov A.A., Miroshnikov K.K., Kabanova A.P., Barannik A.P., Ignatov A.N., Miroshnikov K.A. (2018). Draft genome sequences of new genomospecies “*Candidatus* Pectobacterium maceratum” strains, which cause soft rot in plants. Genome Announc..

[20] Portier P., Pédron J., Taghouti G., Fischer-Le Saux M., Caullireau E., Bertrand C., Laurent A., Chawki K., Oulgazi S., Moumni M. (2019). Elevation of *Pectobacterium carotovorum* subsp. *odoriferum* to species level as *Pectobacterium odoriferum* sp. nov., proposal of *Pectobacterium brasiliense* sp. nov. and *Pectobacterium actinidia*e sp. nov., emended description of *Pectobacterium carotovorum*. Int. J. Syst. Evol. Microbiol..

[21] Dees M.W., Lebecka R., Perminow J.I.S., Czajkowski R., Grupa A., Motyka A., Zoledowska S., Śliwka J., Lojkowska E., Brurberg M.B. (2017). Characterization of *Dickeya* and *Pectobacterium* strains obtained from diseased potato plants in different climatic conditions of Norway and Poland. Eur. J. Plant Pathol..

[22] Malko A., Frantsuzov P., Nikitin M., Statsyuk N., Dzhavakhiya V., Golikov A. (2019). Potato Pathogens in Russia’s Regions: An Instrumental survey with the use of Real-Time PCR/RT-PCR in Matrix Format. Pathogens.

[23] Motyka-Pomagruk A., Zoledowska S., Sledz W., Lojkowska E. (2020). The occurrence of bacteria from different species of Pectobacteriaceae on seed potato plantations in Poland. Eur. J. Plant Pathol..

[24] Ngoc Ha V.T., Voronina M.V., Kabanova A.P., Shneider M.M., Korzhenkov A.A., Toschakov S.V., Miroshnikov K.K., Miroshnikov K.A., Ignatov A.N. (2019). First report of *Pectobacterium parmentieri* causing stem rot disease of potato in Russia. Plant Dis..

[25] Zoledowska S., Motyka A., Zukowska D., Sledz W., Lojkowska E. (2018). Population structure and biodiversity of *Pectobacterium parmentieri* isolated from potato fields in temperate climate. Plant Dis..

[26] Chan B.K., Abedon S.T. (2012). Phage therapy pharmacology. Phage cocktails. Adv. Appl. Microbiol..

[27] Tsonos J., Vandenheuvel D., Briers Y., De Greve H., Hernalsteens J.P., Lavigne R. (2014). Hurdles in bacteriophage therapy: Deconstructing the parameters. Vet. Microbiol..

[28] Evans T.J., Trauner A., Komitopoulou E., Salmond G.P.C. (2010). Exploitation of a new flagellatropic phage of *Erwinia* for positive selection of bacterial mutants attenuated in plant virulence: Towards phage therapy. J. Appl. Microbiol..

[29] Malik D.J., Sokolov I.J., Vinner G.K., Mancuso F., Cinquerrui S., Vladisavljevic G.T., Clokie M.R.J., Garton N.J., Stapley A.G.F., Kirpichnikova A. (2017). Formulation, stabilisation and encapsulation of bacteriophage for phage therapy. Adv. Colloid Interface Sci..

[30] Dees M.W., Lysøe E., Rossmann S., Perminow J., Brurberg M.B. (2017). *Pectobacterium polaris* sp. nov., isolated from potato (*Solanum tuberosum*). Int. J. Syst. Evol. Microbiol..

[31] Staicu L.C., Ackerson C.J., Cornelis P., Ye L., Berendsen R.L., Hunter W.J., Noblitt S.D., Henry C.S., Cappa J.J., Montenieri R.L. (2015). *Pseudomonas moraviensis* subsp. *stanleyae*, a bacterial endophyte of hyperaccumulator *Stanleya pinnata*, is capable of efficient selenite reduction to elemental selenium under aerobic conditions. J. Appl. Microbiol..

[32] Niu B., Paulson J.N., Zheng X., Kolter R. (2017). Simplified and representative bacterial community of maize roots. Proc. Natl. Acad. Sci. USA.

